# Arc hopping dynamics induced by interfacial negative differential resistance

**DOI:** 10.1093/pnasnexus/pgac129

**Published:** 2022-07-25

**Authors:** Jindong Huo, Alex Rontey, Yifei Wang, Linda Jacobs, Qin Chen, Ningzhen Wang, Shilei Ma, Yang Cao

**Affiliations:** Electrical Insulation Research Center, Institute of Materials Science, University of Connecticut, Storrs, CT 06269, USA; Department of Electrical and Computer Engineering, University of Connecticut, Storrs, CT 06269, USA; Electrical Insulation Research Center, Institute of Materials Science, University of Connecticut, Storrs, CT 06269, USA; ABB Industrial Connections & Solutions, Plainville, CT 06062, USA; Applied Materials, Gloucester, MA 01930, USA; Electrical Insulation Research Center, Institute of Materials Science, University of Connecticut, Storrs, CT 06269, USA; Electrical Insulation Research Center, Institute of Materials Science, University of Connecticut, Storrs, CT 06269, USA; Electrical Insulation Research Center, Institute of Materials Science, University of Connecticut, Storrs, CT 06269, USA; Department of Electrical and Computer Engineering, University of Connecticut, Storrs, CT 06269, USA

**Keywords:** negative differential resistance, sheath, arc roots, instability, magnetohydrodynamics

## Abstract

Pattern formation in plasma–solid interaction represents a great research challenge in many applications from plasma etching to surface treatment, whereby plasma attachments on electrodes (arc roots) are constricted to self-organized spots. Gliding arc discharge in a Jacob’s Ladder, exhibiting hopping dynamics, provides a unique window to probe the nature of pattern formation in plasma–surface interactions. In this work, we find that the existence of negative differential resistance (NDR) across the sheath is responsible for the observed hopping pattern. Due to NDR, the current density and potential drop behave as activator and inhibitor, the dynamic interactions of which govern the surface current density re-distribution and the formation of structured spots. In gliding arc discharges, new arc roots can form separately in front of the existing root(s), which happens periodically to constitute the stepwise hopping. From the instability phase-diagram analysis, the phenomenon that arc attachments tend to constrict itself spontaneously in the NDR regime is well explained. Furthermore, we demonstrate via a comprehensive magnetohydrodynamics (MHD) computation that the existence of a sheath NDR can successfully reproduce the arc hopping as observed in experiments. Therefore, this work uncovers the essential role of sheath NDR in the plasma–solid surface pattern formation and opens up a hitherto unexplored area of research for manipulating the plasma–solid interactions.

Significance Statement In the popular science wonder of a Jacob’s ladder, the arc generated inside illustrates fascinating dynamics of movements that feature the hopping of arcs. This perplexing behavior of arc plasma originates from the nonlinear plasma sheath, whereby the potential barrier across features a negative differential resistance (NDR). We conduct stability analysis and magnetohydrodynamic plasma simulation to demonstrate that the NDR-featured sheath potential drop can sufficiently reproduce the arc-root hopping. This finding clarifies the arc hopping behavior originates from the nonlinearity of the sheath instead of the plasma bulk. This work opens a sandbox for manipulating and regulating the plasma–solid interactions, which will be of great interests to both academic and industrial research.

## Introduction

**Fig. 1.**
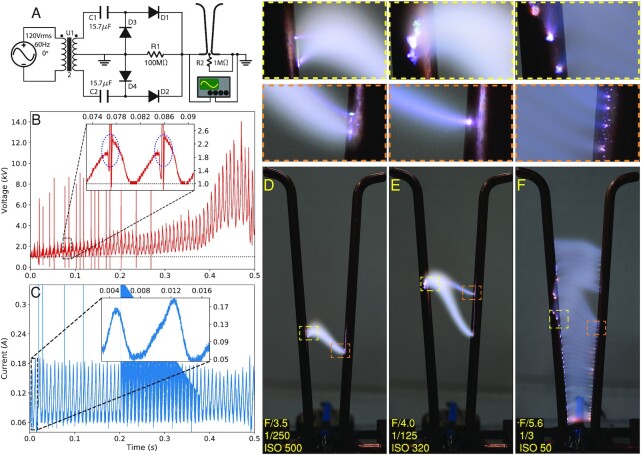
Experimental setup and arc hopping phenomenon. (A) A rectified R–C circuit diagram designed for arc discharge. The resistor R2 is to bridge the electrodes for an easy triggering of the gas discharge. The capacitors C1 and C2, along with the diodes D3 and D4, form two Greinacher voltage doublers allowing for the recovery of the center-tapped transformer’s full peak to peak voltage of up to 20 kV. Diodes D1 and D2 serve to rectify the output seen by the Jacob’s Ladder. The Jacob’s Ladder is made from two copper rods with diameters of 6.2 mm and heights of 186.4 mm. (B) The voltage curve measured by a voltage probe for the whole lifecycle, namely the duration of arc travelling from the bottom to the top. (C) The current waveform measured by a current transformer. Both the current and the voltage have a fundamental frequency of 60 Hz. (D–F) The arc hopping dynamics in long-exposure photos taken by a Sony A7 III camera, with settings (aperture, shutter speed, and sensitivity) marked at the bottom. The magnified views of cathode-arc and anode-arc attachments boxed in yellow and orange, respectively, are shown on the top. Similar arc hopping was also observed in high-current arc discharge (44–46). Please see the video of arc hopping in Movie S3 from the Supplementary Material.

Dynamic arc–solid interactions represent one of the most fundamental, yet challenging research hotspots for a myriad of applications (1). One well-known example is the arc traveling upwards along a “Jacob’s Ladder” with hopping patterns shown in Fig. 1. A huge number of videos of this science wonder can be found on YouTube, which, as taken by STEM (Science, Technology, Engineering and Math) pursuers using a high-speed camera or a camera simply under long-exposure mode, show clearly the arc climbing up the “Jacob’s Ladder” in a stepwise fashion. This intriguing phenomenon of arc hopping has attracted far-reaching attentions, as arcs interaction with solids is widely studied also for many crucial engineering applications such as low-voltage circuit breakers (LVCBs) used in every residential house (2, 3), plasma etching (4), nanomaterial synthesis (5, 6), and biomedical applications (7).

Hardly any existing literatures explain this hopping pattern formation formally, despite gliding arc discharge and arc–electrode interactions have been widely investigated (8–15). A related interpretation argues that the mobility of arc root on electrode was caused by electrode evaporation, which entails arc root instability (9), while some concluded with experiments that arc roots could form within microseconds, which questions the injection flow induced hopping (16). For a physical system, Turing reaction–diffusion model is one of the best known theories to explain self-regulated pattern (17–19), from which pattern forms when two substances, inhibitor and activator, interact with each other. Like in a gas discharge, the charged particles (inhibitor) deposited on the electrode surface inhibit the discharge through their own electric field, but the discharge current (activator) will activate the production of charged particles and itself through the avalanching process of charge multiplication (20, 21) that includes the secondary electron emission. Some studies considered that the negative differential resistance (NDR) played an important role in the pattern formation (22). In gas discharge, a two-layer model, comprising an NDR layer contacting a resistive (Ohmic) layer, has been explored to explain static pattern formations in a dielectric barrier discharge (22–24). However, the existence of NDR in plasma sheath and its effect on dynamic arc–electrode interactions have not been formally investigated.

Plasma sheath forms due to the Debye shielding as the space charge assembles on the wall boundary into a potential barrier that confines the mobile species (25, 26). The potential drop across the sheath, like cathode/anode falls, is spatially nonuniform and is dynamically enhanced in the sheath formation (or attenuated in the sheath dissipation). For example, the field-aligned sheath is annihilated when a heavy electron flux flew through it (27–29). Some studies took advantage of this phenomena by putting forward a current density–dependent potential drop (3, 30) across the sheath. To date, the nonlinear voltage–current characteristics of arc discharge (column) are well recognized (31), while the relation between sheath potential drop and current density remains difficult to measure. In this work, we propose a semi-empirical potential drop model that features NDR. The phenomenon of arc hopping pattern is explained from the perspective of NDR.

In this paper, we first present a gas discharge experiment to illustrate the arc hopping phenomena. Then, we describe the nonuniform potential drop that features NDR. Afterwards, we conduct the stability analysis to study the NDR induced instability at arc attachments. With that, we introduce the diffusion–convection equation and demonstrate the efficacy of NDR in reproducing the arc hopping dynamics with a magnetohydrodynamic (MHD) computer simulation of the arc plasma, resulting good agreement with experiments. This work is expected to improve the understanding of dynamic plasma–surface interactions from the perspective of NDR.

## Results

### Experimental observation of arc hopping

Arc hopping can be readily observed via a gas discharge along a Jacob’s Ladder as shown in Fig. 1 and Movie S1. The bright spots in the long-exposure photos (Fig. 1D–F) correspond to the arc roots. Once initialized, the arc starts to move upwards under the combined effects of thermal buoyancy and Lorentz force. The amplitude of the arc voltage increases as the arc moves upwards as shown in Fig. 1B. After reaching the top of the Jacob’s Ladder, the arc is in the most elongated shape with maximized heat loss due to radiation and conduction, and soon quenches. Examining the electrode surface afterwards reveals no obvious erosion. As shown in Fig. 1C, the discharge circuit is full-wave rectified and there is no polarity reversal induced current zero-crossing to possibly account for the patterned trajectory. The measured discharge voltage fluctuates intermittently around its peak value as shown by the inset in Fig. 1B, due likely to the nonconstant potential drop during arc hopping. This perplexing hopping phenomenon inspires the notion that the plasma-electrode sheath as a bi-stable layer (22) be responsible for the observed hopping.

### Understanding the NDR

Instead of gliding along the electrode surface, the arc roots are observed hopping along the electrodes, indicating certain resistance, which immobilizes arc attachments on the electrode surface, namely the sheath layer. The concept of plasma sheath was initially introduced by Irving Langmuir to describe the interfacial region with a high density of ions that balances the electrons on the wall surface (32). The problem of sheath formation at plasma boundary stands central for nearly all plasma applications, and still remains one of the oldest yet not fully understood problems. The Jacob’s Ladder, a popular science wonder, however, provides a unique “window” to probe the nature of such a mysterious sheath layer.

**Fig. 2.**
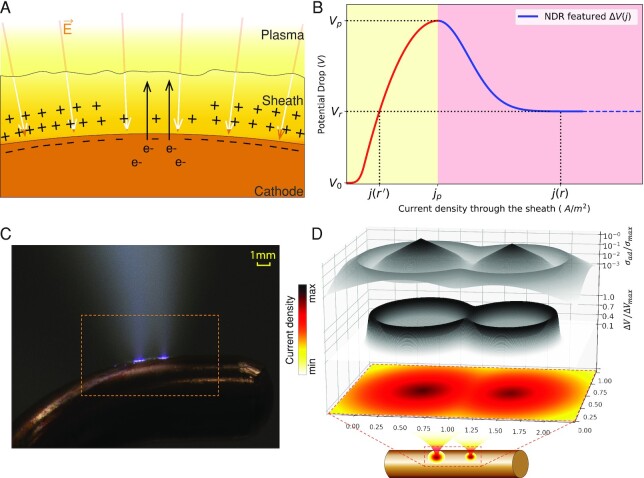
Illustration of sheath potential drop. (A) An illustrative view of space charge configuration in cathode sheath. (B) The ${\boldsymbol{j}} - \Delta {\boldsymbol{V}}$ curve featuring an NDR region posts an ignition voltage (35). ${{\boldsymbol{V}}}_{\boldsymbol{p}}$ is the peak value (ignition voltage) and ${{\boldsymbol{j}}}_{\boldsymbol{p}}$ is the corresponding ignition current density. ${{\boldsymbol{V}}}_{\boldsymbol{r}}$ is the voltage when ${\boldsymbol{j}} \to \infty $. (C) The observation of arc roots. (D) The illustration of nonuniform sheath potential drop. The current density is projected on the bottom plane. On the top are the normalized potential drop $( {\Delta {\boldsymbol{V}}/\Delta {{\boldsymbol{V}}}_{{\boldsymbol{max}}}} )$ and adaptive conductivity (${{\boldsymbol{\sigma }}}_{{\boldsymbol{ad}}}/{{\boldsymbol{\sigma }}}_{{\boldsymbol{max}}}$, where ${{\boldsymbol{\sigma }}}_{{\boldsymbol{ad}}} = {\boldsymbol{\ j}} \cdot \Delta {\boldsymbol{d}}/\Delta {\boldsymbol{V}}$ and $\Delta {\boldsymbol{d}}$ is the sheath thickness). The axis tick labels on the bottom plane indicate the diameter of arc root spots in mm.

With a potential drop across the sheath limiting current (26), strong current shall correspond to a small potential drop (28, 29) as illustrated in Fig. 2A. To continue the seminal but incomplete works (22, 23, 33–36), we thereby consider a potential drop ($\Delta V)$ as a function of current density $( j )$ to capture this subtle relation as shown in Fig. 2B, which features an NDR that has only been implicitly revealed in the literatures (31, 35, 37, 38). Phenomenologically, the surface bright spots with high current density shall have lower potential drop in comparison with the surrounding dark area, indicating a ring-shaped potential barrier as illustrated in Fig. 2C and D.

Because of NDR, the sheath potential drop and the current density behavior as inhibitor and activator, respectively (23). Specifically, the potential drop gives rise to the charge accumulation in the sheath to form a stronger potential barrier, which inhibits the current. Meanwhile, with strong electron emission due to the secondary electron process (28, 39), the discharge current attenuates the potential drop, which in return activates more current density. In a conceivable scenario shown in Fig. 2D, with a local maximum current density ${j}_{max}$ surrounded by a higher potential drop area, the next possible ${j}_{max}$ will have to be located at a distant location.

### A heuristic explanation

To explain arc hopping qualitatively, the arc root and arc column are represented by resistors connected in series, across which the potential drops are ${V}_{r\ }$ and ${V}_{column}$, respectively. For simplification, the arc column is assumed as a regular resistor, while the arc root is a nonlinear resistor. When the surface current increases from zero, the potential drop at the arc root first increases to a peak level (ignition voltage) ${V}_{p\ }$, and then reduces to a low stable value of ${V}_r$ according to Fig. 2B.

Under constant applied voltage, the distribution of current should be such that the total resistance is minimized. If the arc root behaved as a linear resistor, it should have moved upwards smoothly along the electrode surface to follow the arc column, such that the arc can be shortened to reduce its resistance. However, the situation is different if the arc root features NDR.

**Fig. 3.**
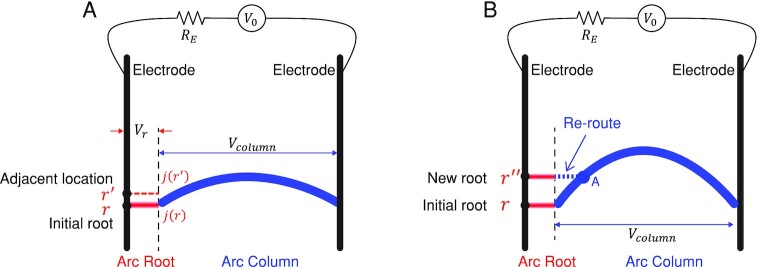
A heuristic model for understanding the arc hopping (considering only the left electrode for simplification). (A) When the arc moved up slightly, with the arc root remaining at its original location. (B) When the arc column moved further, and the potential gap $| {{\boldsymbol{V}}( {\boldsymbol{A}} ) - {\boldsymbol{V}}( {{\boldsymbol{r^{\prime\prime}}}} )} |$ is sufficiently high to re-route the arc to a new root in the front.

Suppose at point ${\boldsymbol{r}}$, the current density $j( {\boldsymbol{r}} )$ is higher than the ignition current density and the potential drop ${V}_{r\ }$ is smaller than ignition voltage as shown in Fig. 3A. Therefore, we can find a point ${\boldsymbol{r}}^{\prime}$ outside the arc root with a current density $j( {{\boldsymbol{r^{\prime}}}} )$ lower than ignition current density but also has the potential drop of ${V}_{r\ }$ as shown in Fig. 2B. However, hardly can the current density at ${\boldsymbol{r^{\prime}}}$ rise further because it enters a very high resistance region. In other words, the arc root tends to be pinned even though the arc column moves upwards slightly.

When the arc continues to elongate, the potential gradient also increases, therefore potential gap between arc column (point A) and front electrode (point ${\boldsymbol{r^{\prime\prime}}}$) finally rises above the ignition voltage as shown in Fig. 3B. Then the arc will “re-route” to a new spot in the front, which completes one step in the hopping.

### Stability analysis

To quantitatively explain how NDR affects the re-distribution of surface current density, we need to investigate the interdependence among three variables in sheath: current density *j*, adaptive conductivity ${\sigma }_{ad}$ ( ${\sigma }_{ad} = \ j \cdot \Delta d/\Delta V$), and potential drop $\Delta V$ (represented by field intensity $E\ = \ \Delta V/\Delta d$, where $\Delta d$ is assumed as a constant thickness). Analysis below will tell whether a given state (${\sigma }_{ad}$, *E, j*) remains stable or becomes unstable if a perturbation occurs.

Based on two primary relations: Ohm’s law (assuming quasi-steady state) and the $j-\Delta V$ relation given by Fig. 2B, we have two equations: $j\ = {\sigma }_{ad}\ \cdot E$ and $\Delta V\ = \ \Delta V( j )$. Thus, ${\sigma }_{ad}$ and *E* can be expressed as a function of *j*, namely $E\ ( j ) = \Delta V( j )/\Delta d\ $ and ${\sigma }_{ad}( j )\ = j/E( j )\ $. The derivatives of ${\sigma }_{ad}$ and *E*, with respect to *j*, can be deduced and recast as functions of themselves as shown in Equation (1). Obviously, Equation (1) describes an autonomous system such that the (${\sigma }_{ad}$, *E*) can be self-driven to change. Any given initial values of (${\sigma }_{ad}$, *E*) with solving Equation (1) can get a phase trajectory. Here, the basic parameter for the stability analysis is current density rather than time, therefore the concept of stable or unstable does not imply a runaway process in time but indicates the stability at a given current density.

(1) $$\begin{eqnarray*}
E^{\prime} &=& \frac{{dE}}{{dj}} = \frac{{d\left( {\Delta V/\Delta d} \right)}}{{dj}} = {f}_2\left( {{\sigma }_{ad},E} \right)\nonumber, \\
\sigma _{ad}^{\prime} &=& \frac{{d{\sigma }_{ad}}}{{dj}} = \frac{{d\left( {j/E} \right)}}{{dj}} = \frac{{1 - {\sigma }_{ad} \cdot {E}^{\prime}}}{E} = {f}_1\left( {{\sigma }_{ad},E} \right).
\end{eqnarray*}
$$

**Fig. 4.**
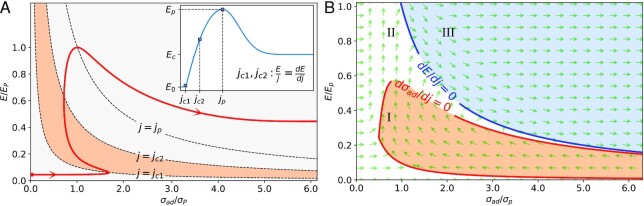
Stability analysis. The coordinates of the phase plane are ${{\boldsymbol{\sigma }}}_{{\boldsymbol{ad}}}$ and ${\boldsymbol{E}}$, which are normalized by dividing ${{\boldsymbol{E}}}_{\boldsymbol{p}}$ and ${{\boldsymbol{\sigma }}}_{\boldsymbol{p}}$, where ${{\boldsymbol{E}}}_{\boldsymbol{p}} = {{\boldsymbol{V}}}_{\boldsymbol{p}}/{\boldsymbol{\Delta d}}$ and ${{\boldsymbol{\sigma }}}_{\boldsymbol{p}} = {{\boldsymbol{j}}}_{\boldsymbol{p}}/{{\boldsymbol{E}}}_{\boldsymbol{p}}$. (A) The red curve is phase trajectory starting from the initial state ( ${{\boldsymbol{\sigma }}}_0 = {\boldsymbol{\ }}0,{\boldsymbol{\ }}{{\boldsymbol{E}}}_0 = {{\boldsymbol{V}}}_0/{\boldsymbol{\Delta d}}$) alone with *j* increasing. The inset represents the ${\boldsymbol{E}}( {\boldsymbol{j}} )$. And ${{\boldsymbol{j}}}_{{\boldsymbol{c}}1}{\boldsymbol{\ and\ }}{{\boldsymbol{j}}}_{{\boldsymbol{c}}2}$ are critical current densities at which ${{\boldsymbol{\sigma }}}_{{\boldsymbol{ad}}}^{\boldsymbol{^{\prime}}} = {\boldsymbol{\ }}0$ (namely ${\boldsymbol{E}}/{\boldsymbol{j}} = {\boldsymbol{dE}}/{\boldsymbol{dj\ at\ j\ }} = {{\boldsymbol{j}}}_{{\boldsymbol{c}}1}{\boldsymbol{\ and\ }}{{\boldsymbol{j}}}_{{\boldsymbol{c}}2}$). The trajectory segment between ${\boldsymbol{j\ }} = {\boldsymbol{\ }}{{\boldsymbol{j}}}_{{\boldsymbol{c}}1}$ and ${\boldsymbol{j\ }} = {\boldsymbol{\ }}{{\boldsymbol{j}}}_{{\boldsymbol{c}}2}$ are deemed stable, because in this segment the change of ${\boldsymbol{j}}$ is partially offset by ${{\boldsymbol{\sigma }}}_{{\boldsymbol{ad}}}$, implying no spontaneous divergence. (B) The nullclines and phase directions. The phase plane is divided into three regions: the red region I is stable; the white region II is transitional; and the blue region III is unstable. Please see Supplementary Material for the method of calculating the phase portrait.

Please see the Supplementary Material for the formulations of ${f}_1$ and ${f}_2$ functions. Due to NDR, $E^{\prime}$ will be negative when current density is high as in the case, where a strong charge flux attenuates the potential drop. An initial state ( ${\sigma }_0 = {\rm{\ }}0,{\rm{\ }}{E}_0 = {V}_0/{\rm{\Delta }}d$) produced phase trajectory is sketched in Fig. 4A. For a general case, we sketch the global nullclines of $d{\sigma }_{ad}/dj\ = \ 0$ and $dE/dj\ = \ 0$ on the phase plane in Fig. 4B. The phase trajectory always follows the phase direction $(\frac{{{f}_1}}{{f_1^2 + f_2^2}}$, $\frac{{{f}_2}}{{f_1^2 + f_2^2}})$ as the green arrows shown in Fig. 4B.

In order to identify the stable or unstable regimes in the phase plane, we first take the variable ${\sigma }_{ad}$ for instance, to explain the stability. Since ${\sigma }_{ad}^{\prime}$ can be rewritten as ${\sigma }_{ad}^{\prime} = \ - \frac{{E^{\prime}}}{E}{\sigma }_{ad} + \frac{1}{E}$, based on Lyapunov’s indirect method, ${\sigma }_{ad}$ possesses the following properties:

- If $E^{\prime}/E > 0$, ${\sigma }_{ad}$ is locally stable under perturbation of $\delta j$.
- If $E^{\prime}/E < 0$, ${\sigma }_{ad}$ is locally unstable under perturbation of $\delta j$.

It is easy to find that $E^{\prime} = \ 0$ is actually a bifurcation condition for ${\sigma }_{ad}^{\prime} = \ - \frac{{E^{\prime}}}{E}{\sigma }_{ad} + \frac{1}{E}$. Please see the Supplementary Figs. S1 and S2 for the plots of the stability analysis variables.

In Fig. 4B, the region I (${\sigma }_{ad}^{\prime} < 0$, $E^{\prime}/E > 0$) is deemed stable, because the variation of the inside state is subject to increasing impedance. For example, the increase of *j* will cause a decrease of ${\sigma }_{ad}$, which prevents *j* from increasing. In this case, the current density is relatively low, and the plasma attachment is likely in a homogeneous state.

The region II (${\sigma }_{ad}^{\prime} > 0$, $E^{\prime}/E > 0$) is a transitional regime, because the increase of *j* will cause the increase of ${\sigma }_{ad}$, which contributes to *j* increasing further, but such increase may not grow.

The region III (${\sigma }_{ad}^{\prime} > 0$, $E^{\prime}/E < 0$) is unstable, because any tiny perturbation $\delta j$ will cause either propagating change of current density. For instance, for a positive perturbation of $+ \delta j$, such $+ \delta j$ will grow continuously as the case of arc roots formation. For a negative perturbation of $- \delta j$, such $- \delta j$ will cause a propagating decay of current density until below ${j}_p$, like in surrounding areas of arc roots.

Due to the sheath contacting the resistive plasma bulk, the formation of arc roots will inevitably constrict the adjacent plasma bulk. Like an NDR resistor connecting a regular resistor in series, the current will be bistable (see negative resistance in Wiki) and depends on the external resistance. Due to gas dynamics, the high temperature in plasma bulk will cause thermal expansion and gas convection, which will give rise to various plasma attachments (40). Thus, a computer simulation is necessary to study the dynamic behavior of arc roots if there is an NDR.

### MHD Simulation

To demonstrate the efficacy of NDR-featured sheath layer for arc hopping, we conduct an MHD simulation that incorporates an NDR sheath layer, in addition to existing plasma modelling approaches (41–43). As one of the governing equations, the charge continuity equation establishes the relation among space charge density ($\rho$), potential field ($\Phi$), and current density (*j*) in Equation (2) which is a typical convection–diffusion equation:

(2) $$\begin{equation*}
\displaystyle\frac{{\partial \rho }}{{\partial t}} + \nabla \cdot \left( {\rho \ \vec{V}} \right) + \nabla \cdot \left( { - \sigma \nabla \Phi } \right) + \nabla \cdot \left( { - \mathop \sum \limits_k {D}_k\nabla {C}_k} \right) = S,
\end{equation*}
$$

where $\vec{V}$ is the flow velocity, *S* is the reaction source, $\sigma $ is the electrical conductivity, ${D}_k$ is the diffusivity, and ${C}_k$ is the product of number density and valence for charged species *k*. If considering the magnetic induction, *S* will include $\nabla \cdot ( { - \sigma \vec{V} \times \vec{B}} )$, where $\vec{B}$ is the magnetic flux density.

If the plasma is quasi-neutral and the flow velocity is low, the convection ($\rho \ \vec{V}$) and diffusion ($- \mathop \sum \limits_k {D}_k\nabla {C}_k$) terms will be negligible compared with the electric conduction ($- \sigma \nabla \Phi $). The conduction term $\nabla \cdot - \sigma \nabla \Phi $ is actually a diffusion in essence, which transports the charges along the electric potential gradient. The source term *S* forces the local generation of charges to counteract the depletion by diffusion. It is worth mentioning that, in the sheath, the electric conductivity (${\sigma }_{ad}$) is not constant and its value depends on local current density. Thus, Equation (2) describes a nonlinear equation.

When a quasi-static state is reached ($\frac{{\partial \rho }}{{\partial t}} \approx 0$), the Equation (2) can be simplified to a pure diffusion–reaction form of $\nabla \cdot \ ( { - \sigma \nabla \Phi } ) = \ S$. In the simulation, the current density–dependent ${\sigma }_{ad}$ is constantly updated in the near-electrode layer to ensure the predefined potential drop following the proposed model defined by Fig. 2B. The MHD simulation needs to solve gas dynamics, heat transfer, and low-frequency electromagnetics. Please see the modelling methods elaborated in the Supplementary Material, simulation model in Supplementary Fig. S3, governing equations in Table S1 and boundary conditions in Table S2.

**Fig. 5.**
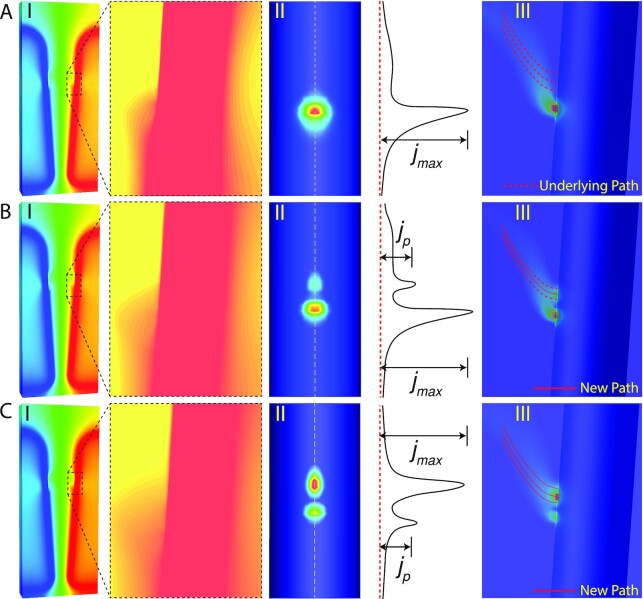
The simulation results of arc hopping at three consecutive moments: before, during, and after the hopping. The color legend is normalized, the blue and red color represent min and max value, respectively. (A–I) The electrical potential field. The color distortion in the zoomed subfigure indicates the nonlinear potential drop across the sheath. (A–II) The top view of current density on the electrode surface. The right-side curve indicates the amplitude of current density along the symmetry plane on the electrode surface. (A–III) The current density distribution before arc hopping. (B, C) The arc root profiles at the subsequent moments during and after the hopping.

The simulation successfully reproduces the arc hopping as shown in Fig. 5 and Movie S2, in which a new arc root forms separately in front of the old one, instead of gliding from it. The arc attachment is always constricted to structured spots, which immobilize itself due to the high potential barrier in the front. This coincides with the aforementioned mechanism of the nonlinear potential barrier surrounding the arc root illustrated in Fig. 2D. The location with higher current density always has a high adaptive conductivity, which accounts for the *constriction* of arc attachments. The magnetic force is considered in the simulation and has a secondary effect on the arc root constriction (please see the results of magnitude pinch in the Supplementary Fig. S4).

Once current density at a remote place fluctuates into NDR regime, it will grow “unstably” to form a new arc root, which soon replaces the existing one, as shown in Fig. 5. If the new arc root is formed before the old one completely dies out, multiple arc roots will coexist. This process happens periodically representing a hopping pattern, which supports the proposed sheath potential drop and NDR sheath. On the other hand, we find the arc hopping would completely vanish if the simulation used a constant potential drop, i.e. no NDR effect. Please see the Supplementary Figs. S5 and S6 for the illustration of NDR effect on current density re-distribution.

## Summary

Travelling arc along a Jacob’s Ladder showcases a fascinating hopping pattern. It is suggested in this study that the existence of NDR in plasma sheath be responsible for such an arc hopping, demonstrated by both analytical analysis and MHD simulation. Due to NDR, the positive perturbation of current density will activate discharge current that contributes to the arc roots formation. Meanwhile, the high potential drop surrounding the arc root behaves as an inhibitor for the discharge current and immobilizes the arc attachments. Due to gas dynamics, a new arc attachment can form at a distant location, toward which the arc column bends forward. In a further demonstration, the MHD arc simulation incorporated with an NDR-featured near-electrode layer reproduces the arc hopping as observed in experiments. Without NDR, the arc hopping pattern would completely vanish. This finding reveals the existence of NDR across plasma sheath that gives rise to the dynamic arc hopping, which furthers the understanding of pattern formation in plasma–solid interactions in a broad perspective.

## Supplementary Material

pgac129_Supplemental_File

## Data Availability

All data are included in the manuscript and/or Supplementary Material.
