# Supplementary material for: Arc hopping dynamics induced by interfacial negative differential resistance
Source: PNAS Nexus. 2022 Jul 25;1(3):pgac129. doi: 10.1093/pnasnexus/pgac129 (PMC9896960; doi:10.1093/pnasnexus/pgac129)
Supplement: pgac129_Supplemental_File [file pgac129_supplemental_file.zip › PNASNEXUS-PNASNEXUS-2022-00395-s03.pdf]

PNAS nexus

Supplementary Information for

Arc hopping dynamics induced by interfacial negative differential resistance

Jindong Huo[1], Alex Rontey[2], Yifei Wang[1], Linda Jacobs[3], Qin Chen[4], Ningzhen Wang[1], Shilei Ma[1], Yang Cao[1,2*]

*Corresponding author: Yang Cao
Email: yang.cao@uconn.edu

This PDF file includes:

Supplementary text

Figures S1 to S6

Tables S1 to S2

SI References 1 to 34

Other supplementary materials for this manuscript include the following:

Movies S1 to S2

Supplementary Information Text

1 Stability analysis

1.1 Explicit function formulation

Without loss of generality, the plasma and its attachment on the electrode shall be referred to as "arc" and "arc root", respectively. For the current-density dependent potential drop across the sheath, the potential drop featuring a negative differential resistance (NDR) is shown in Fig. 2B. We will demonstrate that NDR in potential drop plays a crucial role in the structured plasma attachments.

In order to quantify the NDR effect, the explicit expression for the potential drop $\Delta V = \Delta V(j)$ is formulized as a piecewise function as shown in Equation S1. This expression ensures the $\Delta V(j)$ and its first order derivative are continuous. The real $V(j)$ function is difficult to decide and expected to depart from the expression given for an illustrative purpose, but the conclusion should be similar as long as NDR exists.

$$\Delta V(j) = \begin{cases} V_0 + C_0 j^4 & [0, j_0) \\ V_p - C_1 (j - j_p)^2 & [j_0, j_p) \\ (V_p - V_c) e^{-\frac{(j-j_p)^2}{C_2}} + V_c & [j_p, \infty) \end{cases} \tag{S1}$$

where the V_0 is the initial potential drop when $j \to 0$; V_p is the peak/ignition potential drop and V_c is the potential drop when $j \to \infty$; the coefficient C_0 and C_1 are defined as $C_0 = \frac{V_p - V_0}{{j_0}^3 (j_p - j_0/2)}$, $C_1 = \frac{V_p - V_0}{(j_p - j_0)(j_p - j_0/2)}$ to ensure $\Delta V(j)$ and $d\Delta V/dj$ are continuous. The parameters, j_0, j_p, V_0, V_p and V_c, are case-dependent, and some of them have been reported [1-6] . For example, V_0 should be a value close to the work function of the electrode material. When $j = j_p$, the potential drop reaches its maximum. For the copper electrodes, we assume $j_0 \approx 10^3\ A/m^2$, $j_p \approx 10^4 A/m^2$, $V_0 \approx 0.1 V_c$, $V_p \approx 2.25 V_c$ (V_c is the potential drop across cathode sheath).

The electric field intensity E inside the sheath is calculated as $E = \Delta V/\Delta d$ where Δd is the sheath thickness (a constant). Thus, E is a function of j, and its derivative E' is:

$$E'(j) = \frac{dE}{dj} = \begin{cases} 4C_0 j^3/\Delta d & [0, j_0) \\ -2C_1(j - j_p)/\Delta d & [j_0, j_p) \\ -\frac{2(j - j_p)(E_p - E_c)}{C_2} \cdot e^{-\frac{(j-j_p)^2}{C_2}} & [j_p, \infty) \end{cases} \tag{S2}$$

where $E_p = V_p/\Delta d$; $E_c = V_c/\Delta d$. When $j = j_p$, $E'(j_p) = 0$. For stability analysis, by replacing $j\ by\ \sigma_{ad} \cdot E$, the $E'(j)$ can be expressed as the function of σ_{ad} and E, which is:

$$E' = f_2(\sigma_{ad}, E) = \begin{cases} 4C_0(\sigma_{ad}E)^3/\Delta d & [0, j_0) \\ -2C_1(\sigma_{ad}E - j_p)/\Delta d & [j_0, j_p) \\ -\frac{2(\sigma_{ad}E - j_p)(E_p - E_c)}{C_2} \cdot e^{-\frac{(\sigma_{ad}E - j_p)^2}{C_2}} & [j_p, \infty) \end{cases} \tag{S3}$$

The adaptive conductivity is defined as $\sigma_{ad} = j/E(j)$, thus $\sigma_{ad}' = \frac{d\sigma_{ad}}{dj} = \frac{d(j/E)}{dj} = \frac{E - jE'}{E^2} = \frac{1 - \sigma_{ad}E'}{E}$. By introducing Equation S3 into $\sigma_{ad}' = \frac{1 - \sigma_{ad}E'}{E}$, then σ_{ad}' is rewritten as:

$$\sigma_{ad}{}' = f_1(\sigma_{ad}, E) = \frac{1-\sigma_{ad}E'}{E} = \begin{cases} \frac{1}{E} - 4C_0\sigma_{ad}{}^4E^2/\Delta d & [0, j_0) \\ \frac{1}{E} + 2C_1\left(\sigma_{ad}{}^2 - \frac{\sigma_{ad}\cdot j_p}{E}\right) & [j_0, j_p) \\ \frac{1}{E} + \frac{2\sigma_{ad}(\sigma_{ad}E - j_p)(E_p - E_c)}{c_2 E} e^{-\frac{(\sigma_{ad}E - j_p)^2}{c_2}} & [j_p, \infty) \end{cases} \tag{S4}$$

So far, the explicit expressions of $f_1(\sigma_{ad}, E)$ and $f_2(\sigma_{ad}, E)$ are obtained as Equations S3 and S4. The explicit expressions of $E(j)$, $\sigma_{ad}(j)$ and their derivatives E' and $\sigma_{ad}{}'$ are calculated and plotted in Fig.S*1*. The $E(j)$ curve in Fig.S*1*(A) is the same as the inset in Fig.4A in the main text.

In the main text, it mentions that the convergence of σ_{ad} is decided by E'. Similarly, the convergence of E is decided by $\sigma_{ad}{}'$ as well. Specifically, since $E = j/\sigma_{ad}$, E' can be expressed as:

$$E' = \frac{dE}{dj} = \frac{d(j/\sigma_{ad})}{dj} = \frac{\sigma_{ad} - j\sigma_{ad}}{\sigma_{ad}{}^2} = -\frac{\sigma_{ad}{}'}{\sigma_{ad}} \cdot E + \frac{1}{\sigma_{ad}} \tag{S5}$$

Thus E possesses the following properties:

- if $\frac{\sigma_{ad}{}'}{\sigma_{ad}}$<0, the variation of E is locally unstable.
- if $\frac{\sigma_{ad}{}'}{\sigma_{ad}}$>0, the variation of E is locally stable.

1.2 Phase trajectory

The property of $\sigma_{ad}{}'$ largely dominates the distribution of current density at arc roots. For example, the increase of σ_{ad} will cause the increase of current density, which in return further enhances the σ_{ad} especially at the NDR regime and then activates more current density, which means a given state (σ_{ad}, E) is unstable if $\sigma_{ad}{}' > 0$.

From Fig.S*2* (A), $j = j_{c2}$ is a critical current density where $\sigma_{ad}{}' = 0$. For the low current density case when $j < j_{c2}$, the sheath can be regarded as a regular resistor and arc attachments will be in a homogeneous state. For high current density case when $j > j_{c2}$ (especially for the NDR regime $j > j_p$), the sheath is a nonlinear resistor which causes the spontaneous change of current density.

The trajectories starting from different initial conditions share similar features, as plotted on Fig.S*2* (B), that E reaches its maxima at $j = j_p$.

Due to the NDR, $+\delta j$ gives rise to a propagating increase of current density; and $-\delta j$ will cause a propagating decrease of current density. The NDR in the sheath is regarded as the sufficient cause for the arc hopping. Please see the supplementary code for the calculating and plotting the phase trajectory and diagram.

1.3 The diffusion-reaction equation

As discussed in the main text, for low-frequency arc plasma, the charge continuity equation will be simplified into a reaction-diffusion form, which is always employed in the study of self-regulated pattern formation. The reaction-diffusion models are normally in the forms of $\frac{\partial Y}{\partial t} = \nabla \cdot (D\nabla Y) + S(Y)$, in which *D* is the diffusivity, $\frac{\partial Y}{\partial t}$ represents the transient term, $\nabla \cdot (D\nabla Y)$ represents the diffusion term and $S(Y)$ represents the reactive/source term. If $S(Y)$ can be expanded as a function of the state variable Y, namely $S(Y) = \mu Y + \alpha|Y|^2Y + o(Y)$, this

equation is of the Ginzburg-Landau type[7], which describes the evolution of physical variables in unstable modes exhibiting a Hopf bifurcation.

Generally, a dynamic system of reaction-diffusion can be regarded as the competition between the production, depletion(diffusion) process of Y [7]. For example, the source term $S(Y)$ will force the generation of Y, and the diffusion term will transport Y into the low concentration/potential location and thus decide its distribution. When a steady state is reached, which means $\frac{\partial Y}{\partial t} = 0$, the global equilibration between the production and depletion, namely $\nabla \cdot (-D\nabla Y) = S(Y)$, will be reached and give rise to a structured pattern or homogeneous distribution.

Mathematically speaking, the electric conduction, $\nabla \cdot (-\sigma\nabla\Phi)$, is actually a diffusion in essence, which is induced by the potential gradient instead of the concentration gradient. For the equation (2) in the main text, the diffusion term has a special property such that the diffusivity σ in the sheath layer is nonlinear and depends on the diffusion flux (in other words, the σ depends on $j = -\sigma\nabla\Phi$ and even has $d\sigma/dj > 0$). Due to this special property, the aforementioned equilibration may not be stable, and only certain kinds of current density distribution (and potential structure) will be stable, namely the structured spots mode or a homogeneous mode. For the reactive source term in Equation (2), we consider the source induced by magnetic induction $\nabla \cdot \left(-\sigma\vec{V} \times \vec{B}\right)$ which is the divergence of induction current density. The complex evolution of these variables can be completed by the computer simulation.

2 Simulation method

The charge continuity equation (2) in the main text is the governing equation that determines the distribution of current density in the near-electrode layer. After the electrical potential (Φ) is obtained, the current density can be calculated as $j = -\sigma\nabla\Phi + \sigma\vec{V} \times \vec{B}$. The following is about the simulation method used to obtain the results in Fig.4 and Movie S2.

2.1 Geometry and mesh

In the simulation, the sheath is treated as a spatial layer, within which the adaptive conductivity is employed to ensure a predefined potential drop therein[8, 9]. The adaptive conductivity is constantly updated by local current density in the sheath, to maintain the Fig.2B relation between potential drop and charge flux (component of current density normal to the interface). The sheath thickness refers to Debye length[10] $\lambda_D = \left(\frac{\varepsilon_0 K T_e}{ne^2}\right)^{1/2}$, in which T_e is the electron temperature; n is the electron number density; K is Boltzmann constant; e is electron charge; ε_0 is vacuum permittivity. The simulation domain is 90mm long, 30mm thick and 220mm high. Considering the high computational expense, we adopt a constant sheath thickness for approximation ($\approx 0.05mm$). Due to the instability of NDR, it requires adopting a fine mesh for the near-electrode layer in the simulation as shown in Fig.S3 (C).

From many attempts, the free mesh (like tetrahedral mesh) compromises the calculation of the diffusive flux through the elements interface. Thus, a structured mesh is suggested for solving the charge continuity equation, which is shown in Fig.S3.

2.2 Simplification and assumption

To avoid unnecessary complexity, the transport properties of the plasma mixture are calculated and tabulated prior to the simulation [9, 11]. For high pressure plasma, it is assumed that the collision dominates the collective behavior, such that the energy gained from the electric field are timely equilibrated by the collision among particles. Thus, the arc plasma can be appropriately described by continuum thermodynamic/fluid models. We postulate the quasi-neutrality for the arc column [12]. Since the permittivity is very small ($\varepsilon \approx 10^{-12} F/m$) and frequency is low, the effect of displacement current ($\varepsilon\, \partial \boldsymbol{E}/\partial t$) on magnetic field is ignored in the simulation. The simulation considers the self-induced magnetic pinch by adding a source term in the momentum equation ($\vec{J} \times \vec{B}$). Significant progress has been made in modelling arc plasma through magnetohydrodynamics (MHD) [13-17]. Modelling the arc plasma incorporates solving fluid dynamics, radiation/conduction heat transfer, electromagnetic fields, governed by the Navier-Stokes's equations, radiative transfer equation and electromagnetic equations, respectively.

Since the temperature near the electrodes is lower than that in the arc bulk, the hypothesis of local thermal equilibrium (LTE) gives rise to significantly low electrical conductivity in the near-electrodes region [18]. Due to charge emission and ambipolar diffusion, the near-electrode regions are actually conductive[19]. The deviation from LTE is embodied by considering two temperatures for electrons and heavy particles, respectively[20]. The simulation results are obtained by numerically solving the transient-advection-diffusion-reaction equation (S6):

$$\frac{\partial(\rho\Phi)}{\partial t} + \nabla \cdot \left(\rho \vec{V} \Phi\right) = \nabla \cdot (\Gamma_\Phi \nabla \Phi) + S_\Phi \qquad (S6)$$

where $\partial(\rho\Phi)/\partial t$ represents the transient term; $\nabla \cdot (\rho\vec{V}\Phi)$ is the advective term; $\nabla \cdot (\Gamma_\Phi \nabla\Phi)$ is the diffusive term and S_Φ represents the reactive/source term. $\vec{V}$ is the flow velocity. The computation is performed via a finite volume solver.

In Table S*1*, μ is the viscosity coefficient, g is the gravitational acceleration, $\bar{\bar{\boldsymbol{\tau}}}$ is the viscous stress. h_e is electron enthalpy defined as $h_e = \frac{5}{2} K_B n_e T_e/\rho$ [21, 22], where n_e is the electron number density; h_h is the enthalpy for heavy particles defined as $h_h = \frac{5}{2}\frac{k_B}{\rho}\sum_{i=1}^{N} n_i \left(T_h + E_i + T_h^2 \frac{\partial ln Q_i^{int}}{\partial T_h}\right)$. If the specific heat $C_p = \frac{\partial h_h}{\partial T}$ [22, 23] is introduced, the enthalpy can be rewritten as $h_h = \int C_p dT_h$. The electron energy source due to the density gradient is $-\nabla \cdot \left(\frac{5}{2} k_B T_e \vec{J}_{\boldsymbol{D,e}}\right)$ where $\vec{J}_{\boldsymbol{D,e}} = -D_e \nabla \mathrm{n_e}$ and $\mathrm{n_e}$ is the number density of electron, D_e is the electron diffusivity[24]. The energy exchange between the electron and heavy particle is Q_{e-h} (please see reference [19]). In the charge continuity equation, given the Poisson equation ($\rho = \varepsilon \nabla \cdot \vec{E}$) and $\vec{E} = -\nabla\Phi$, the transient term $\frac{\partial \rho}{\partial t}$ can be recast as $\frac{\partial}{\partial t}(-\varepsilon \nabla^2 \Phi)$. At quasi-static state, the transient term $\frac{\partial \rho}{\partial t}$ is close to zero and is ignored in the simulation. Besides, due to the characteristic length for the simulation is much larger than Debye length, the continuum-based simulation averages out the variations within the scale of Debye length.

To supplement the existing method for modelling arc dynamics[16, 19, 27, 28], the sheath is implemented as a spatial layer rather than a 1D interface. Specifically, the adaptive conductivity (σ_{ad}) is employed in the sheath layer to ensure the current-density-dependent potential drop. In addition, we consider the heat flux imposed on

both cathode and anode spots (arc attachments) in addition to the conjugate heat transfer. Please see reference [25] for more details.

2.3 Boundary conditions

The boundary conditions include pressure boundary (considering buoyancy), wall boundary, current in/out, exterior cathode/anode wall, internal gas-metal interface, and symmetry boundary. The symmetry boundary condition is applied to the symmetry plane, and the other boundary conditions are summarized in Table S*2* [9, 17, 19, 20, 30]. The coupled boundary condition means the variable is continuous across the interface inside the computation domain. The boundary types listed in Table S*2* are marked accordingly in Fig.S3 (A).

At the beginning, an empirical arc is initialized by giving the predefined temperature and pressure fields. After that, two time-steps calculation is performed to initialize the electromagnetic fields, during which the fluid equations are frozen to ensure the self-consistency between electromagnetic fields and plasma thermal state (temperature and pressure). Then, all equations are coupled and solved.

2.4 Additional simulation results

As expected, the simulation achieves the hopping dynamics of arc roots by the discontinuous displacement of arc attachments as shown in Fig. 5 and Fig.S4(A). The maximum value of magnetic pinch $(\vec{f} = \vec{j} \times \vec{B})$ and the maximum current density are shown in Fig.S4(B). Please see the simulation results in Movies S2.

3 Pattern formation at arc attachments

The sheath layer, plasma bulk and electrodes are connected in series, which can be regarded as an NDR resistor connecting PDR (positive differential resistance) resisters as illustrated in Fig.S5(A). Because of NDR, there will be a bistable region for the circuit current in each branch (see https://en.wikipedia.org/wiki/Negative_resistance). The arc attachments can be regarded as a parallel circuit with infinite branches, and thus the bistable current in each branch will together contribute to multiple-stable modes of current density distribution as illustrated in Fig.S5(B). The MHD simulation provides a unique tool to test and illustrate the NDR effects on the pattern formation at arc roots.

Under low current discharge condition (like grow discharge), the current density distribution at the attachments is in a diffuse mode, and its temporal evolution is subject to self-increasing impedance (i.e., the increase of j will cause the decrease of σ_{ad} which prevent the j from increasing, and vice versa), as illustrated in Fig.S6(B). Once the maximum current density exceeds the j_p, the state (E, σ_{ad}, j) will be unstable. For example, under controlled discharge current, if the maximum current density is at attachment center, the conductivity at the center will also be largest and will continue to increase, which will make the attachments constricts further. Thus, the spot becomes more and more conspicuous as shown in Fig.S6(C). Apart from the interfacial NDR, it has been reported that the arc attachment modes are affected by factors like the thermal state of electrodes, the amplitude of discharge current and electrical conductivity of electrodes[7, 31-33], therefore the spatial-temporal evolution of pattern formation at arc attachments needs further study.

4 Codes for calculating and plotting phase diagram

Below is Python3 code used to calculate and plot the phase trajectory (Fig4A in the main text).

```
import matplotlib
import numpy as np
import matplotlib.pyplot as plt
from scipy.integrate import odeint

## run in python 3
######################################################   Define the E, dS/dj, dE/dj function
thickness=0.0001
Ec=20/thickness  # the value matters little, if normalized
E0=0.1*Ec
Ep=2.25*Ec
j0, jp, jf=1e3, 1e4, 2e4

c0=(Ep-E0)/(j0**3*(2*jp-j0))
c1=(Ep-E0)/((jp-j0)*(jp-j0/2))
c2=(jf-jp)**2.0/np.log(1000)

def E_field(j):
    if j<0.0:
        return 'Incorrect current density'
    elif j<=j0:
        return c0*j**4+E0
    elif j<=jp:
        return -c1*(j-jp)**2+Ep
    else:
        return (Ep-Ec)*np.exp(-(j-jp)**2.0/c2)+Ec

def dSdj(sigma, E):
    jtempj2=sigma*E
    if jtempj2<=j0:
        return 1/E-4*c0*jtempj2**4/E**2
    elif jtempj2<=jp:
        return 1/E+2*c1*jtempj2*(jtempj2-jp)/E**2
    else:
        return 1/E+2*jtempj2*(jtempj2-jp)*(Ep-Ec)/(c2*E**2)*np.exp(-(jtempj2-jp)**2/c2)

def dEdj(sigma, E):
    jtemp=sigma*E
    if jtemp<=j0:
        return 4*c0*jtemp**3
    elif jtemp<=jp:
        return 2*c1*(jp-jtemp)
    else:
        return 2*(Ep-Ec)*(jp-jtemp)/c2*np.exp(-(jtemp-jp)**2/c2)

####################################################################  solve PDE
def ESigma(y, j):
    sigma, E = y
    dydt = [dSdj(sigma, E), dEdj(sigma, E)]
    return dydt
jlist=np.linspace(10, 3.0e4, num=400)
y0=[10/E_field(10), E_field(10)]
solution = odeint(ESigma, y0, jlist)

sigma_jplot=np.linspace(1e-4, 0.14, num=200)
sigma_jplot1=np.linspace(1e-4, 0.050, num=100)
sigma_jplot2=np.linspace(0.064, 0.15, num=100)

jc1=0.70*j0  # approximate value
jc2=0.45*jp

E_jc1=[jc1/i for i in sigma_jplot]
E_jc2=[jc2/i for i in sigma_jplot]
E_jp =[jp/i  for i in sigma_jplot]

##############################################################################  Plot Figure
fig,ax=plt.subplots(figsize=(8,5))
ax.plot(sigma_jplot1, [jc1/i for i in sigma_jplot1], 'k--',  linewidth=1.0)
ax.plot(sigma_jplot1, [jc2/i for i in sigma_jplot1], 'k--', linewidth=1.0)
ax.plot(sigma_jplot1, [jp/i for i in sigma_jplot1], 'k--', linewidth=1.0)

ax.plot(sigma_jplot2, [jc1/i for i in sigma_jplot2], 'k--', linewidth=1.0)
ax.plot(sigma_jplot2, [jc2/i for i in sigma_jplot2], 'k--', linewidth=1.0)
ax.plot(sigma_jplot2, [jp/i for i in sigma_jplot2], 'k--', linewidth=1.0)

ax.fill_between(sigma_jplot, E_jc1, E_jc2, alpha=0.5, edgecolor='none', facecolor='#FF9848', linewidth=1,
linestyle='dashed', antialiased=True)
```

```
ax.text(0.057, 1.2*jc1/0.057, r"$j=j_c1$", size=16, rotation=0.0, ha="center", va="center")
ax.text(0.057, jc2/0.057, r"$j=j_c2$", size=16, rotation=0.0, ha="center", va="center")
ax.text(0.057, jp/0.057, r"$j=j_p$", size=16, rotation=0.0, ha="center", va="center")

ax.scatter([10/E_field(10)], [E_field(10)], s=18,  marker='s', color='r', edgecolor='r', label='Tradjactory',
linewidth=1.2)
ax.plot(solution[:, 0], solution[:, 1], 'r-', label='Tradjactory', linewidth=2.5)

for nn in [2, 240]:
    XX, YY=solution[nn:nn+1, 0], solution[nn:nn+1, 1]
    U=solution[nn:nn+1, 0]-solution[nn-1:nn, 0]
    V=solution[nn:nn+1, 1]-solution[nn-1:nn, 1]
    U2 = U / np.sqrt(U**2 + V**2)
    V2 = V / np.sqrt(U**2 + V**2)
    Q=ax.quiver(XX, YY, U2, V2, units='width', width=0.003, headwidth=10, headlength=10, scale_units='width',
scale=30, angles='xy', color='r')

sigma_p=jp/Ep
ax.set_xlabel('$\sigma/ \sigma_P$', color='k', fontsize=14, fontname='sans-serif')
ax.set_ylabel('$ E/E_P  $', color='k', fontsize=14, fontname='sans-serif')

ax.set_xticks(     [round(a,3) for a in np.arange(0, 6.1*sigma_p, sigma_p)])
ax.set_xticklabels([round(a/sigma_p,1) for a in np.arange(0, 6.1*sigma_p, sigma_p)], fontsize=14, fontname='sans-
serif')
ax.set_yticks(     [round(a,4)    for a in np.arange(0.0*Ep, 1.1*Ep, Ep/5)])
ax.set_yticklabels([round(a/Ep,1) for a in np.arange(0.0*Ep, 1.1*Ep, Ep/5)], fontsize=14, fontname='sans-serif')

ax.set_xlim(0, 6.2*sigma_p)
ax.set_ylim(0, 1.2*Ep)

plt.show()
plt.close()
plt.clf()
```

Below is Python3 code used to calculate and plot the phase diagram (Fig4B in the main text).

```
import matplotlib
import numpy as np
import matplotlib.pyplot as plt
from scipy.integrate import odeint

# run in Python3
#######################################################   E, dS/dj, dE/dj function

thickness=0.0001
Ec=20/thickness   # this value matters little, if normalized
E0=0.1*Ec
Ep=2.25*Ec
j0, jp, jf=1e3, 1e4, 2e4


c0=(Ep-E0)/(j0**3*(2*jp-j0))
c1=(Ep-E0)/((jp-j0)*(jp-j0/2))
c2=(jf-jp)**2.0/np.log(1000)

def E_field(j):
    if j<0.0:
        return 'Incorrect current density'
    elif j<=j0:
        return c0*j**4+E0
    elif j<=jp:
        return -c1*(j-jp)**2+Ep
    else:
        return (Ep-Ec)*np.exp(-(j-jp)**2.0/c2)+Ec

def dSdj(sigma, E):
    jtempj2=sigma*E
    if jtempj2<=j0:
        return 1/E-4*c0*jtempj2**4/E**2
    elif jtempj2<=jp:
        return 1/E+2*c1*jtempj2*(jtempj2-jp)/E**2
    else:
        return 1/E+2*jtempj2*(jtempj2-jp)*(Ep-Ec)/(c2*E**2)*np.exp(-(jtempj2-jp)**2/c2)
```

```
def dEdj(sigma, E):
    jtemp=sigma*E
    if jtemp<=j0:
        return 4*c0*jtemp**3
    elif jtemp<=jp:
        return 2*c1*(jp-jtemp)
    else:
        return 2*(Ep-Ec)*(jp-jtemp)/c2*np.exp(-(jtemp-jp)**2/c2)

##############################################################################  dE/dj=0  dS/dj=0
c, r=201, 226    # resolution
Slist=np.linspace(0.00001, 0.15, c)
Elist=np.linspace(0.001, 0.6e6, r)

SS, EE=np.meshgrid(Slist, Elist)
SStemp, EEtemp=SS.reshape(r*c), EE.reshape(r*c)
dSdJ2D, dEdJ2D, jj=np.array([]), np.array([]), np.array([])

for l, k in zip(SStemp, EEtemp):
    dSdJ2D=np.append(dSdJ2D, dSdj(l,k))
dSdJ2D=dSdJ2D.reshape(r,c)

for m, n in zip(SStemp, EEtemp):
    dEdJ2D=np.append(dEdJ2D, dEdj(m,n))
dEdJ2D=dEdJ2D.reshape(r,c)

#############################################################################  phase arrow
column, row=24, 24
Sigma = np.linspace(0, 0.15, num=column)
E = np.linspace(100, 0.7e6, num=row)

X, Y=np.meshgrid(Sigma, E)
Xtemp=X.reshape(row*column)
Ytemp=Y.reshape(row*column)
dE_dSx, dE_dSy=np.array([]), np.array([])

for i,j in zip(Xtemp,Ytemp):
    dE_dSx=np.append(dE_dSx, dSdj(i,j))
    dE_dSy=np.append(dE_dSy, dEdj(i,j))

dE_dSx=dE_dSx.reshape(row,column)
dE_dSy=dE_dSy.reshape(row,column)

dE_dSx2 = dE_dSx/np.sqrt(dE_dSx**2 + dE_dSy**2)
dE_dSy2 = dE_dSy/np.sqrt(dE_dSx**2 + dE_dSy**2)

#############################################################################   Plot Figure
fig,ax=plt.subplots(figsize=(8,5))
cs1 = ax.contour(SS, EE, dEdJ2D, levels=[0], colors='b', linestyles='-', linewidths=2.5)
cs2 = ax.contour(SS, EE, dSdJ2D, levels=[0], colors='r', linestyles='-', linewidths=2.5)

p = cs2.collections[0].get_paths()[0]
v = p.vertices
xtest, ytest = v[:,0], v[:,1]
cracknum=int(0.415*len(v))     # annotation location

labelpos=[(0.031, 2e5)]
ax.clabel(cs1, cs1.levels, inline =True, fmt={0:'$dE/dj=0$'}, fontsize=18, manual=labelpos)
ax.clabel(cs2, cs2.levels, inline =True, fmt={0:'$d \sigma_{ad} /dj=0 $'}, fontsize=18,
manual=labelpos)

ax.fill_between(xtest[cracknum:], 0, ytest[cracknum:], alpha=0.4, facecolor='tab:orange',
linewidth=1, linestyle='dashed', antialiased=True)
ax.fill_between(xtest[0:cracknum], 0, ytest[0:cracknum], alpha=1, facecolor='w', linewidth=1,
linestyle='dashed', antialiased=True)
ax.fill_between(Slist, [jp/i for i in Slist], 2*Ep,    alpha=0.3, facecolor='cornflowerblue',
linewidth=1, linestyle='dashed', antialiased=True)

ax.text(0.016, 0.16e6, r'I', size=20, rotation=0.0, ha="center", va="center", fontname='Times New
Roman', color='k')
```

```
ax.text(0.017, 0.38e6, r'II', size=20, rotation=0.0, ha="center", va="center", fontname='Times New Roman', color='k')
ax.text(0.045, 0.38e6, r'III', size=20, rotation=0.0, ha="center", va="center", fontname='Times New Roman', color='k')

Q = ax.quiver(X, Y, dE_dSx2, dE_dSy2, units='width', width=0.0025, headwidth=6, headlength=6, scale_units='width', scale=36, angles='xy', color='g', alpha=0.5)

ax.set_xlabel('$\sigma/ \sigma_{p}$', color='k', fontsize=14, fontname='sans-serif')
ax.set_ylabel('$ E/E_{p} $', color='k', fontsize=14, fontname='sans-serif')

sigma_p=jp/Ep
ax.set_xticks(     [round(a,4) for a in np.arange(0, 7*sigma_p, sigma_p)])
ax.set_xticklabels([round(a/sigma_p,1) for a in np.arange(0, 7*sigma_p, sigma_p)], fontsize=14, fontname='sans-serif')

ax.set_yticks(     [round(a,4) for a in np.arange(0, 1.1*Ep, 0.2*Ep)])
ax.set_yticklabels([round(a/Ep,1) for a in np.arange(0, 1.1*Ep, 0.2*Ep)], fontsize=14, fontname='sans-serif')

ax.set_xlim(0.0, 0.14)
ax.set_ylim(0, 0.46e6)

plt.show()
plt.close()
plt.clf()
```

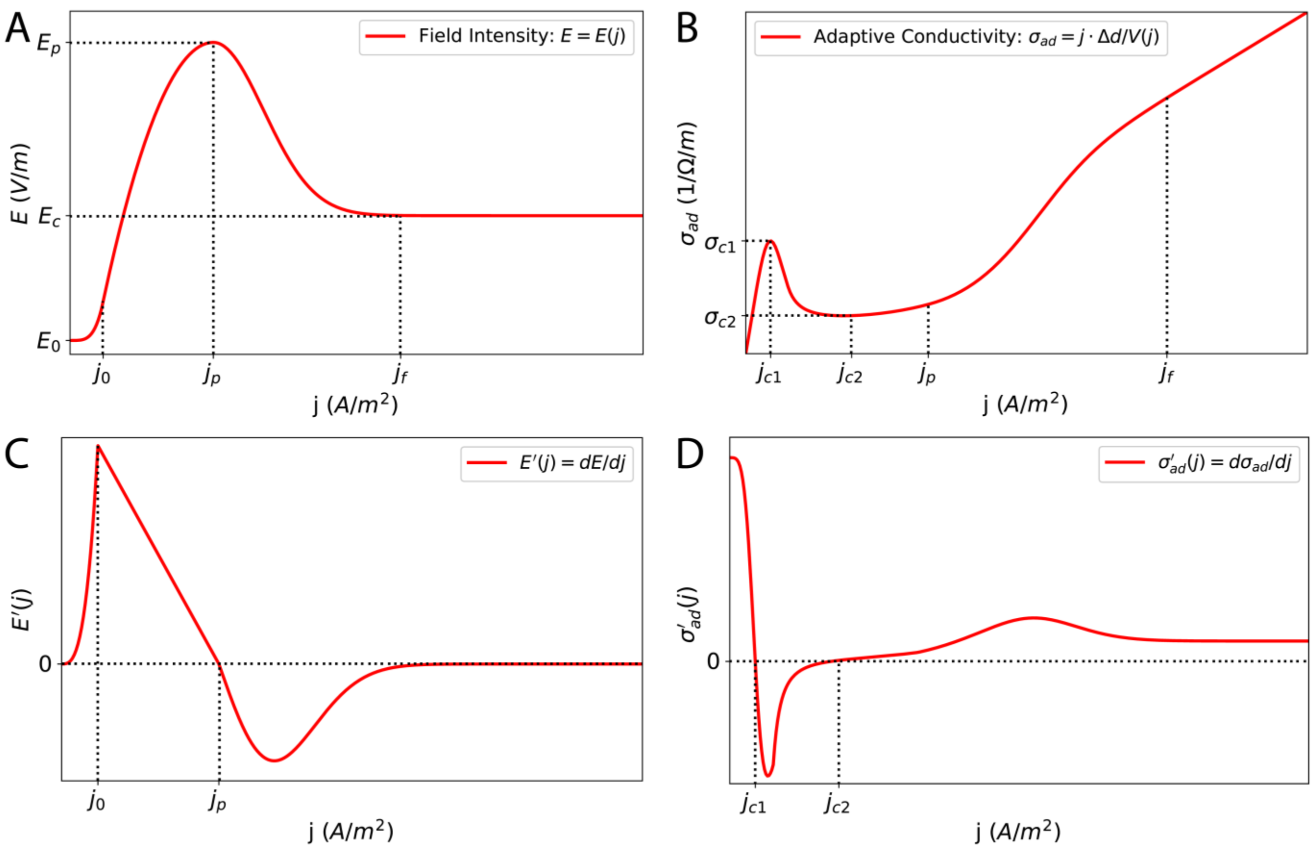

Fig.S1. The initial condition ($\sigma_{ad} = \sim 0, E = V_0/\ \Delta d$) at $j = 0$ together with solving Equation S3 and S4 gives the explicit expressions of $E = E(j), \sigma_{ad} = \sigma_{ad}(j)$ as plotted in (A) and (B). The corresponding derivative functions: $E^{'}(j) = dE/dj, \sigma_{ad}{}^{'}(j) = d\sigma_{ad}/dj$ are plotted in (C) and (D), respectively.

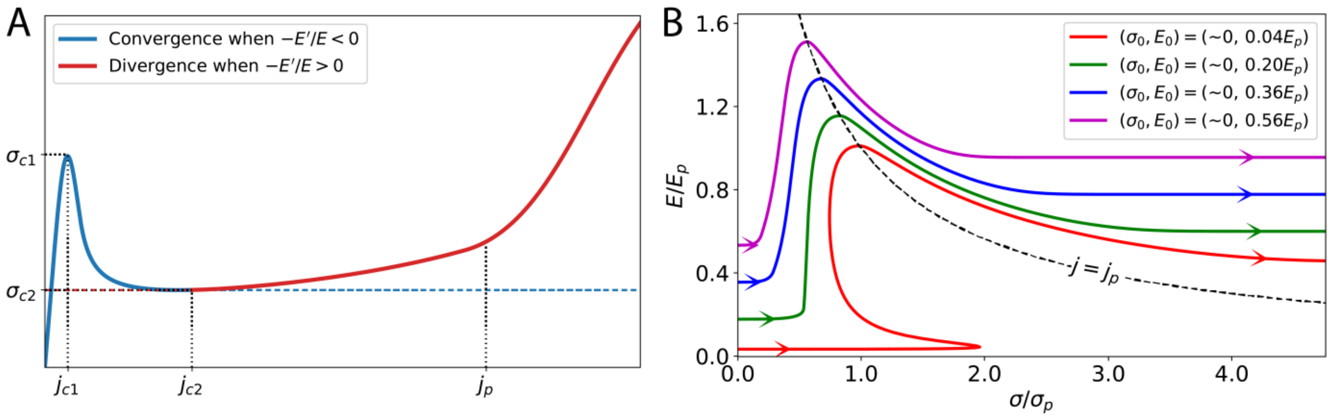

Fig.S2. Phase trajectories. (A) The convergence criterion for σ_{ad}. When $A < 0$, $\sigma_{ad}{}' = A\sigma_{ad} + C$ is stable as the blue curve shows; When $A > 0$, $\sigma_{ad}{}' = A\sigma_{ad} + C$ is unstable as the red curve shows. The tick labels for Y axis are $\sigma_{c1} = \sigma_{ad}(j_{c1}), \sigma_{c2} = \sigma_{ad}(j_{c2})$. (B) The phase trajectory starting from different initial conditions show high similarity.

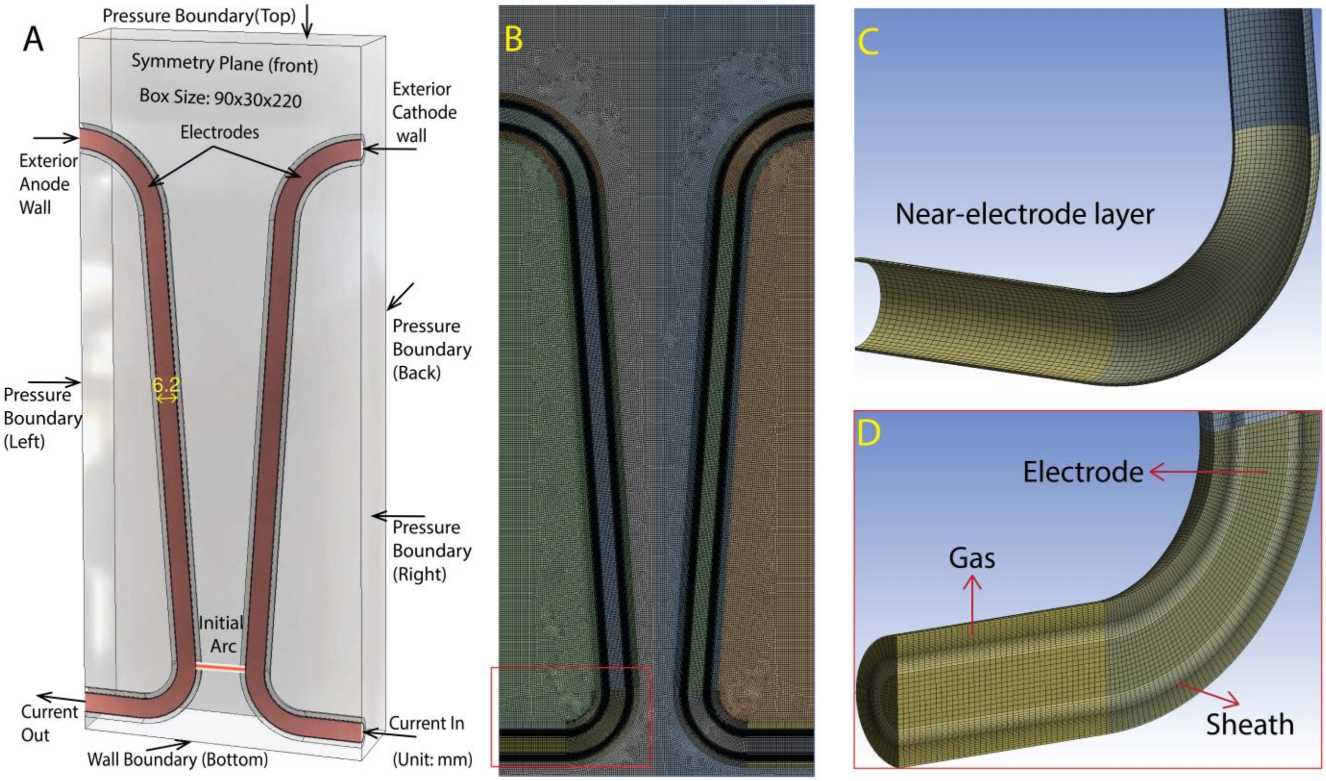

Fig.S3. Simulation model. (A) The geometry is plane symmetric and consists of two copper rods. Please note the sheath is very thin layer (~0.05mm) covering the electrode in the figure (this is intentionally created for structured mesh). (B) The mesh, which is consisted of hexahedron elements. The mesh is densified on both sides of the near-electrode layer (sheath) to capture the large gradients of field variables, like temperature and electrical potential. (C, D) The densely meshed electrode and near-electrode layer (including the sheath).

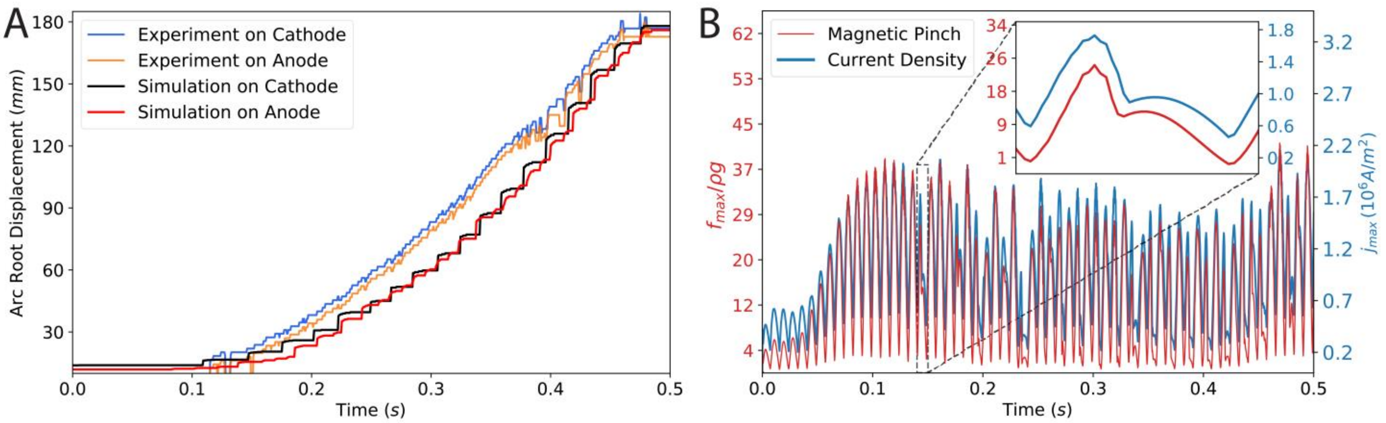

Fig.S4 Simulation results. (A) The comparison of arc root displacements between experiment and simulation. The arc root is identified as the location of maximum current density in the simulation, and most bright point in the experimental observation (obtained by image pixel analysis by MATLAB). (B) The time evolution of maximum current density j_{max} and maximum magnetic pinch force f_{max} at arc root. The axis label represents the ratio of maximum pinch to gravity ($f_{max}/\rho g$).

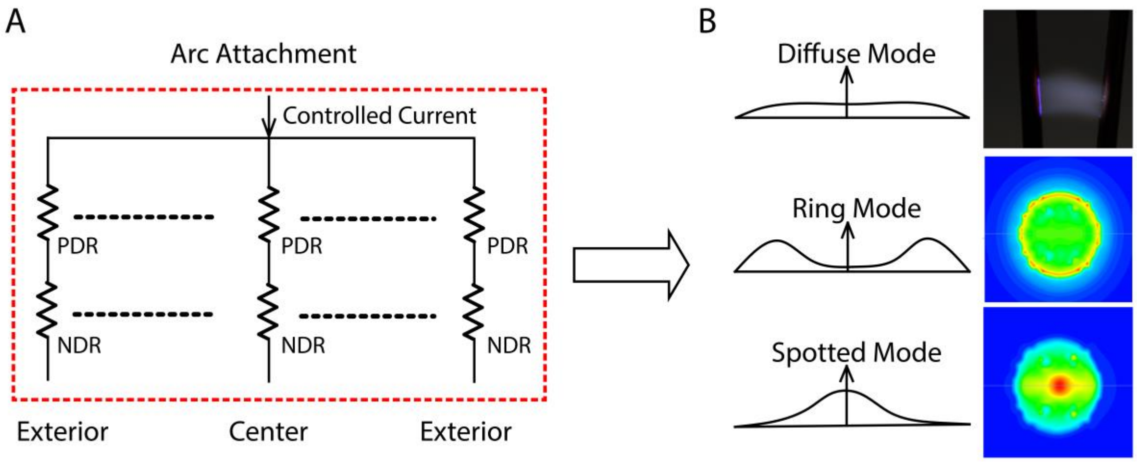

Fig.S5 The NDR featured sheath layer and its effect on current density re-distribution. (A) The analogy of arc attachments by a circuit system. Each branch has a bistable region for circuit current. (B) The possible modes of current density distribution and pattern formation at arc attachments (the contours are obtained by simulation).

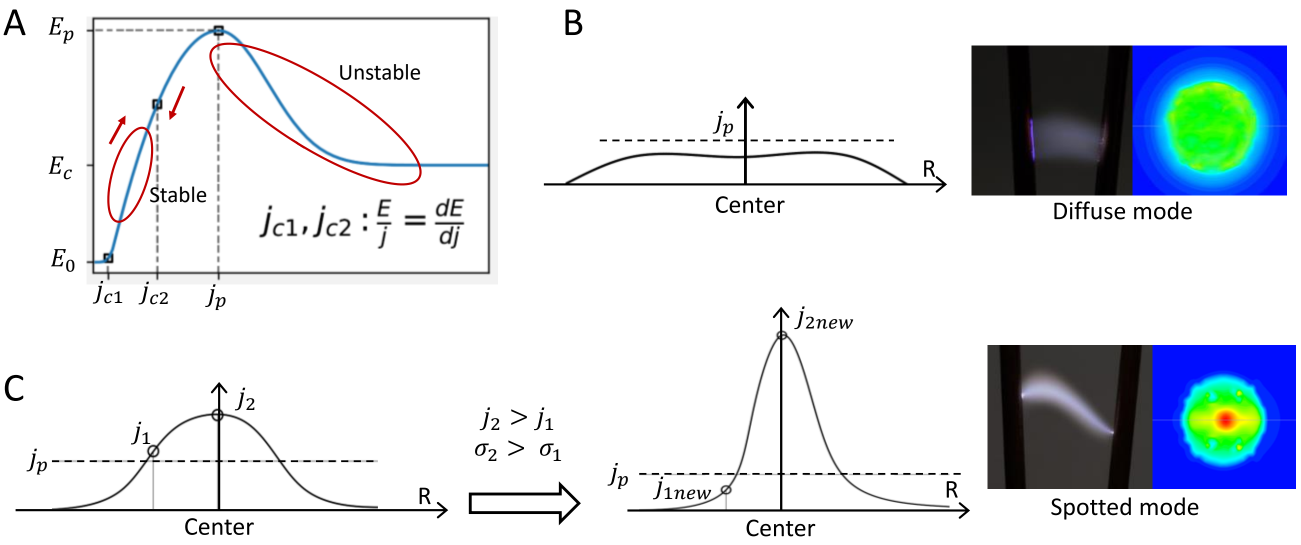

Fig.S6 The temporal evolution of current density distribution at arc attachments. (A) The illustration of $j - E$ curve, namely the $j - \Delta V/\Delta d$ curve. The segment between j_{c1} and j_{c2} is deemed stable, inside which the current density variation is subject to increasing impedance. When $j > j_p$, any tiny perturbation of δj will grow spontaneously. (B) For an arc attachment, if the maximum current density is smaller than j_p, the distribution will be likely in a diffusing mode, no NDR effect. (C)For a constant applied total current, If the maximum current density j_2 exceeds j_p at the attachment center, the center will have highest σ_{ad} (even a higher σ'_{ad}), therefore the current density re-distribution will evolve into a more conspicuous spot.

Table S1. Fluid electromagnetic equations for arc plasma (Transient + Advective = Diffusive + Source)

Equation	Transient	Advective	Diffusive	Reactive/Source
Conservation of mass	$\frac{\partial\rho}{\partial t}$	$\nabla\cdot(\rho\vec{V})$	0	0
Conservation of momentum X component	$\frac{\partial(\rho V_x)}{\partial t}$	$\nabla\cdot(\rho V_x\vec{V})+\frac{\partial P}{\partial x}$	$\nabla\cdot(\mu\nabla V_x)+\nabla\cdot(\mu\frac{\partial\vec{V}}{\partial x})-\frac{\partial}{\partial x}(\frac{2}{3}\mu\nabla\cdot\vec{V})$	$j_yB_z-j_zB_y$
Conservation of momentum Y component	$\frac{\partial(\rho V_y)}{\partial t}$	$\nabla\cdot(\rho V_y\vec{V})+\frac{\partial P}{\partial y}$	$\nabla\cdot(\mu\nabla V_y)+\nabla\cdot(\mu\frac{\partial\vec{V}}{\partial y})-\frac{\partial}{\partial y}(\frac{2}{3}\mu\nabla\cdot\vec{V})$	$j_zB_x-j_xB_z-g$
Conservation of momentum Z component	$\frac{\partial(\rho V_z)}{\partial t}$	$\nabla\cdot(\rho V_z\vec{V})+\frac{\partial P}{\partial z}$	$\nabla\cdot(\mu\nabla V_z)+\nabla\cdot(\mu\frac{\partial\vec{V}}{\partial z})-\frac{\partial}{\partial z}(\frac{2}{3}\mu\nabla\cdot\vec{V})$	$j_xB_y-j_yB_x$
Energy of heavy particles	$\frac{\partial(\rho h_h)}{\partial t}$	$\nabla\cdot(\rho h_h\vec{V})$	$\nabla\cdot(k_h\nabla T_h)$	$-P_h\nabla\cdot\vec{V}+\bar{\bar{\boldsymbol{\tau}}}:\nabla\vec{V}+Q_{e-h}$
Energy of electron	$\frac{\partial(\rho h_e)}{\partial t}$	$\nabla\cdot(\rho h_e\vec{V})$	$\nabla\cdot(k_e\nabla T_e)$	$-Q_{e-h}+\frac{5k_B}{2e}\vec{j}\cdot\nabla T_e+\vec{j}\cdot\vec{E}+$ $-\nabla\cdot\left(\frac{5k_B}{2}T_e\vec{j}_{D,e}\right)+Q_{rad}$
Energy Electrode	$\frac{\partial(\rho_s C_v T)}{\partial t}$	0	$\nabla\cdot(k\nabla T)$	$\vec{j}^{\mathbf{2}}/\sigma_s$
Charge Continuity	$\frac{\partial\rho}{\partial t}$	0	$\nabla\cdot(\sigma\nabla\Phi)$	$\nabla\cdot(-\sigma\vec{V}\times\vec{B})$
Magnetic Vector Potential component (A_x)	0	0	∇^2A_x	$-\mu_0j_x$
Magnetic Vector Potential component (A_y)	0	0	∇^2A_y	$-\mu_0\,j_y$
Magnetic Vector Potential component (A_z)	0	0	∇^2A_z	$-\mu_0j_z$

Table S2. Boundary Conditions

Variables	*Pressure Boundary (Top)*	*Pressure Boundary (Left, Right, Back)*	*Bottom Wall*	*Current In*	*Current Out*	*Exterior Anode Cathode wall*	*Internal Gas-Metal Interface*
Pressure	*Static Pressure*	*Floating Pressure:* $p_s - \rho g h$	$\frac{\partial P}{\partial \vec{n}} = 0$	--	--	--	$\frac{\partial P}{\partial \vec{n}} = 0$
V_x	$\frac{\partial V_x}{\partial \vec{n}} = 0$	$\frac{\partial V_x}{\partial \vec{n}} = 0$	*0*	--	--	--	$V_x = \vec{V_s} \cdot \vec{e}_x$
V_y	$\frac{\partial V_y}{\partial \vec{n}} = 0$	$\frac{\partial V_y}{\partial \vec{n}} = 0$	*0*	--	--	--	$V_y = \vec{V_s} \cdot \vec{e}_y$
V_z	$\frac{\partial V_z}{\partial \vec{n}} = 0$	$\frac{\partial V_z}{\partial \vec{n}} = 0$	*0*	--	--	--	$V_z = \vec{V_s} \cdot \vec{e}_z$
Temperature (Heavy Particle)	$T_h = RT$ *if backflow. Else* $\frac{\partial T_h}{\partial \vec{n}} = 0$	$T_h = RT$ *if backflow. Else* $\frac{\partial T_h}{\partial \vec{n}} = 0$	$T_h = RT$	$T_h = RT$	$T_h = RT$	$T_h = RT$	$Coupled$
Temperature (Electron)	$\frac{\partial T_e}{\partial \vec{n}} = 0$	$\frac{\partial T_e}{\partial \vec{n}} = 0$	$\frac{\partial T_e}{\partial \vec{n}} = 0$	$--$	$--$	--	$Balanced$
A_x	$\frac{\partial A_x}{\partial \vec{n}} = 0$	$\frac{\partial A_x}{\partial \vec{n}} = 0$	$\frac{\partial A_x}{\partial \vec{n}} = 0$	$\frac{\partial A_x}{\partial \vec{n}} = 0$	$\frac{\partial A_x}{\partial \vec{n}} = 0$	$\frac{\partial A_x}{\partial \vec{n}} = 0$	$Coupled$
A_y	$\frac{\partial A_y}{\partial \vec{n}} = 0$	$\frac{\partial A_y}{\partial \vec{n}} = 0$	$\frac{\partial A_y}{\partial \vec{n}} = 0$	$\frac{\partial A_y}{\partial \vec{n}} = 0$	$\frac{\partial A_y}{\partial \vec{n}} = 0$	$\frac{\partial A_y}{\partial \vec{n}} = 0$	$Coupled$
A_z	$\frac{\partial A_z}{\partial \vec{n}} = 0$	$\frac{\partial A_z}{\partial \vec{n}} = 0$	$\frac{\partial A_z}{\partial \vec{n}} = 0$	$\frac{\partial A_z}{\partial \vec{n}} = 0$	$\frac{\partial A_z}{\partial \vec{n}} = 0$	$\frac{\partial A_z}{\partial \vec{n}} = 0$	$Coupled$
Φ	$\frac{\partial \Phi}{\partial \vec{n}} = 0$	$\frac{\partial \Phi}{\partial \vec{n}} = 0$	$\frac{\partial \Phi}{\partial \vec{n}} = 0$	*Current Input:* $\vec{j} = I/A$	*Earth Grounded:* $\Phi = 0$	$\frac{\partial \Phi}{\partial \vec{n}} = 0$	$Coupled$
Radiation	*Transparent* $\tau = 100\%$	*Transparent* $\tau = 100\%$	*Semi-transparent* $\rho = 80\%$ $\tau = 20\%$	--	--	--	*Opaque* $\alpha = 100\%$

The meanings of the symbols used in the Table S*2* are:

Φ : Electrical potential
T_e : Electron temperature
ρ : Radiation Reflectivity
α : Radiation Absorptivity
τ : Radiation Transmissivity
$\vec{e}_x, \vec{e}_y, \vec{e}_z$: Unit vector on X, Y, Z direction, respectively
T_h : Temperature of heavy particles
$\vec{V_s}$: Stefan flow velocity (if no evaporation, its value is zero)
$\vec{n}$: Unit vector normal to the boundary/interface
$\vec{j}$ *and* I : Current density and arc current amplitude.

Movie S1

The video of arc hopping dynamics in a Jacob's Ladder was taken by a high-speed camera, Photron FASTCAM SA5, with a frame rate of 3000 fps. The Movie S1 includes a slow-motion of the real case. The total duration time is ~0.6 second. The left branch is the anode. The right branch is the cathode.

The simulation results are shown in the second half. The legend indicates the amplitude of current density. For the simulation model, the left branch of Jacob's ladder is the cathode with a fixed electrical potential of zero as would be the case if it were earth grounded.

(A high-resolution video has been uploaded to YouTube: https://youtu.be/Sf0DgYZt5yM)

Movie S2

Another video (YouTube: https://youtu.be/JPsB9NefGRY) records the arc hopping in a high-current discharge experiment, where multiple arc roots coexist on the electrode surfaces.

SI References

[1] J.M. Lafferty, Vacuum arcs: Theory and applications, John Wiley & Sons1980.
[2] C. Charles, A review of recent laboratory double layer experiments, Plasma Sources Science and Technology 16(4) (2007) R1-R25.
[3] A. Mutzke, T. Rüther, M. Lindmayer, M. Kurrat, Arc behavior in low-voltage arc chambers, The European Physical Journal-Applied Physics 49(2) (2010) 22910.
[4] A. Khrabry, I.D. Kaganovich, V. Nemchinsky, A. Khodak, Investigation of the short argon arc with hot anode. II. Analytical model, Physics of Plasmas 25(1) (2018) 013522.
[5] C. Kong, J. Gao, J. Zhu, A. Ehn, M. Aldén, Z. Li, Characterization of an AC glow-type gliding arc discharge in atmospheric air with a current-voltage lumped model, Physics of Plasmas 24(9) (2017) 093515.
[6] P. Flesch, M. Neiger, Numerical simulation of dc high-pressure discharge lamps including electrodes, Journal of Physics D: Applied Physics 35(14) (2002) 1681.
[7] J.P. Trelles, Pattern formation and self-organization in plasmas interacting with surfaces, Journal of Physics D: Applied Physics 49(39) (2016).
[8] A. Mutzke, T. Ruther, M. Kurrat, M. Lindmayer, E.-D. Wilkening, Modeling the arc splitting process in low-voltage arc chutes, Electrical contacts-2007, the 53rd ieee holm conference on, IEEE, 2007, pp. 175-182.
[9] J. Huo, S. Selezneva, L. Jacobs, Y. Cao, Study of wall ablation on low-voltage arc interruption: The effect of Stefan flow, Journal of Applied Physics 125(21) (2019) 213302.
[10] F.F. Chen, Introduction to plasma physics and controlled fusion, Springer1984.
[11] J.O. Hirschfelder, C.F. Curtiss, R.B. Bird, M.G. Mayer, Molecular theory of gases and liquids, Wiley New York1954.
[12] L.P. Block, A double layer review, Astrophysics and Space Science 55(1) (1978) 59-83.
[13] J.P. Trelles, C. Chazelas, A. Vardelle, J.V.R. Heberlein, Arc Plasma Torch Modeling, Journal of Thermal Spray Technology 18(5-6) (2009) 728-752.
[14] M.S. Benilov, Understanding and modelling plasma–electrode interaction in high-pressure arc discharges: a review, Journal of Physics D: Applied Physics 41(14) (2008) 144001.
[15] S.A. Al-Mamun, Y. Tanaka, Y. Uesugi, Two-Temperature Two-Dimensional Non Chemical Equilibrium Modeling of Ar–CO2–H2 Induction Thermal Plasmas at Atmospheric Pressure, Plasma Chemistry and Plasma Processing 30(1) (2009) 141-172.
[16] M. Baeva, R. Kozakov, S. Gorchakov, D. Uhrlandt, Two-temperature chemically non-equilibrium modelling of transferred arcs, Plasma Sources Science and Technology 21(5) (2012) 055027.
[17] M. Baeva, D. Loffhagen, D. Uhrlandt, Unified Non-equilibrium Modelling of Tungsten-Inert Gas Microarcs in Atmospheric Pressure Argon, Plasma Chemistry and Plasma Processing 39(6) (2019) 1359-1378.
[18] R. Huang, H. Fukanuma, Y. Uesugi, Y. Tanaka, Comparisons of two models for the simulation of a DC arc plasma torch, Journal of thermal spray technology 22(2-3) (2013) 183-191.
[19] M. Baeva, Non-equilibrium modeling of tungsten-inert gas arcs, Plasma Chemistry and Plasma Processing 37(2) (2017) 341-370.
[20] P. Freton, J.J. Gonzalez, Z. Ranarijaona, J. Mougenot, Energy equation formulations for two-temperature modelling of ‘thermal’ plasmas, Journal of Physics D: Applied Physics 45(46) (2012) 465206.
[21] J.P. Trelles, Computational study of flow dynamics from a dc arc plasma jet, Journal of Physics D: Applied Physics 46(25) (2013) 255201.
[22] W.Z. Wang, M.Z. Rong, J.D. Yan, A.B. Murphy, J.W. Spencer, Thermophysical properties of nitrogen plasmas under thermal equilibrium and non-equilibrium conditions, Physics of Plasmas 18(11) (2011) 113502.
[23] V. Colombo, E. Ghedini, P. Sanibondi, Thermodynamic and transport properties in non-equilibrium argon, oxygen and nitrogen thermal plasmas, Progress in Nuclear Energy 50(8) (2008) 921-933.
[24] A.A. Bobrov, A.M. Bunkov, S.Y. Bronin, A.B. Klyarfeld, B.B. Zelener, B.V. Zelener, Conductivity and diffusion coefficients in fully ionized strongly coupled plasma: Method of molecular dynamics, Physics of Plasmas 26(8) (2019) 082102.
[25] J. Huo, Y. Wang, Y. Cao, 3D computational study of arc splitting during power interruption: the influence of metal vapor enhanced radiation on arc dynamics, Journal of Physics D: Applied Physics 54(8) (2020) 085502.
[26] J. Haidar, A theoretical model for gas metal arc welding and gas tungsten arc welding. I, Journal of Applied Physics 84(7) (1998) 3518-3529.
[27] G. Xu, J. Hu, H.L. Tsai, Three-dimensional modeling of the plasma arc in arc welding, Journal of Applied Physics 104(10) (2008) 103301.

[28] F. Yang, M. Rong, Y. Wu, A.B. Murphy, J. Pei, L. Wang, Z. Liu, Y. Liu, Numerical analysis of the influence of splitter-plate erosion on an air arc in the quenching chamber of a low-voltage circuit breaker, Journal of Physics D: Applied Physics 43(43) (2010) 434011.
[29] J. Hu, H.L. Tsai, Heat and mass transfer in gas metal arc welding. Part I: The arc, International Journal of Heat and Mass Transfer 50(5-6) (2007) 833-846.
[30] B. Swierczynski, J.J. Gonzalez, P. Teulet, P. Freton, A. Gleizes, Advances in low-voltage circuit breaker modelling, Journal of Physics D: Applied Physics 37(4) (2004) 595.
[31] C. Chazelas, J.F. Coudert, P. Fauchais, Arc root behavior in plasma spray torch, IEEE Transactions on Plasma Science 33(2) (2005) 416-417.
[32] T. Verreycken, P. Bruggeman, C. Leys, Anode pattern formation in atmospheric pressure air glow discharges with water anode, Journal of Applied Physics 105(8) (2009).
[33] J.P. Trelles, Formation of self-organized anode patterns in arc discharge simulations, Plasma Sources Science and Technology 22(2) (2013) 025017.
